# Congenital Visceral Vascular Variation Causing Gastrointestinal Hemorrhage: A Case Report

**DOI:** 10.3389/fped.2021.772529

**Published:** 2021-11-18

**Authors:** Yingli Wei, Zhiqiu Ye, Ning Shang, Chaoxiang Yang, Minyan Liao, Yunxiang Pan

**Affiliations:** ^1^Department of Diagnosis Ultrasound, Guangdong Women and Children Hospital, Guangzhou, China; ^2^Department of Invasive Technology, Guangdong Women and Children Hospital, Guangzhou, China; ^3^Department of Radiology, Guangdong Women and Children Hospital, Guangzhou, China

**Keywords:** hepatic artery, celiac trunk, blood, vessel, variation

## Abstract

Variations in the visceral vasculature are often encountered, but rarely cause clinical symptoms. We report a 12-year-old girl with portal hypertension caused by congenital variations in visceral vessels. The clinical manifestations included gastrointestinal hemorrhage and ascites. The common hepatic artery and splenic artery stem shared the same trunk from the aorta, and the common hepatic artery was directly connected with the main portal vein to form an arteriovenous fistula. In addition, the left hepatic artery and the left gastric artery shared a common trunk termed the “hepatic-gastric trunk” which originated from the anterior wall of the aorta, while the right hepatic artery originated from the superior mesenteric artery and supplied the right liver. The patient was treated with interventional embolization and remained in good condition throughout the follow-up and at the time of publication.

## Introduction

Congenital variation of the visceral vessels, especially those concerning the celiac trunk and hepatic artery, is well-documented in the medical literature (1–3). However, not all variations have been described, and some may be difficult to diagnose in clinical practice. This causes great challenges to surgeons and interventional radiologists when dealing with abdominal vascular diseases (4, 5). Accurate identification of these vascular variations is an important basis for the selection of clinical treatment options, improves the probability of successful surgery, and reduces post-operative complications.

## Case Report

This study was approved by the Ethics Committee of Guangdong Women and Children Hospital. The patient's mother provided written informed consent prior to the treatment procedures.

A 12-year-old girl was admitted with hematemesis and melena. She had no history of chronic liver disease or inherited diseases. The physical examination revealed a heart rate of 99 bpm, blood pressure 89/55 mmHg, and the skin mucosa was pale. Laboratory data showed that hemoglobin was 67 g/L, stool occult blood test (+); and electrolyte, kidney and coagulation function, serum amylase, and liver function tests were normal. The abdomen was flat and soft, with varicose abdominal wall, and without tenderness or rebound pain.

**Figure 1:**
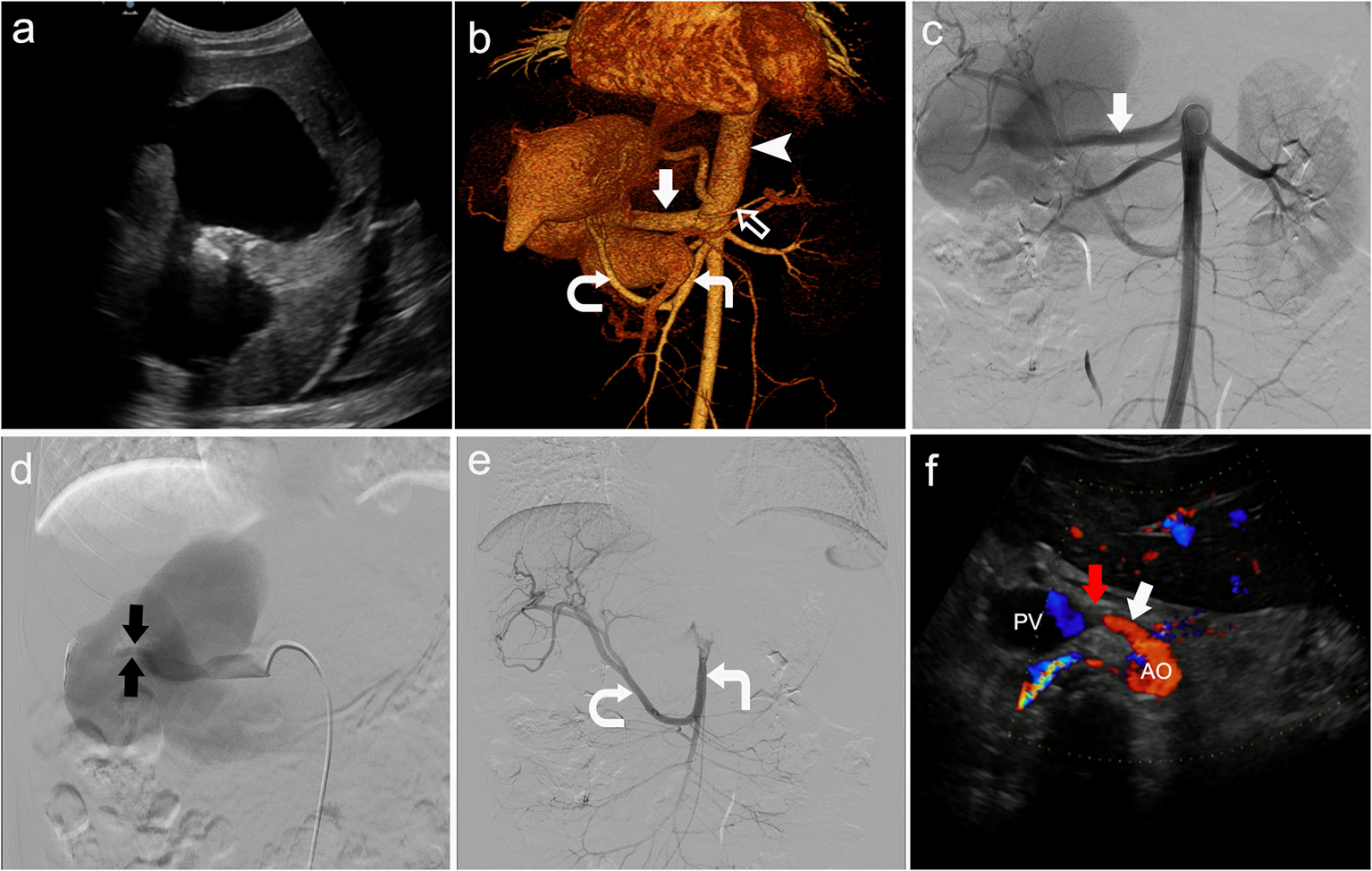
Congenital visceral vascular variation was diagnosed by multi-imaging examinations. **(a)** Tumor-like expansion of the main portal vein and the left branch of the portal vein shown by color Doppler ultrasound. **(b)** Contrast-enhanced computed tomography scan showed the left hepatic artery (black arrow) and left gastric artery (not shown) co-trunk, as the first branch of the aorta (arrowhead). The common hepatic artery (straight solid arrow) and splenic artery (hollow arrow) originated from the front wall of the aorta below the left hepatic-gastric trunk, while the right hepatic artery (curved arrow) originated from the superior mesenteric artery (right angle arrow). **(c)** Angiogram showed the common hepatic artery (straight solid arrow). **(d)** Angiogram showed the fistula (the tiny blood vessels between the two black arrows). **(e)** Angiogram showed that the right hepatic artery (curved arrow) originated from the superior mesenteric artery (right angle arrow). **(f)** The position of the occluder (red arrow) was normal and the fistula was completely closed, 3 months after surgery.

Color Doppler ultrasound revealed a common trunk of the common hepatic artery and splenic artery originating from the anterior wall of the aorta, and the common hepatic artery was directly merged into the portal vein (Figures 1b–e). There was a tumor-like expansion of the main portal vein and the left branch of the portal vein, which were about 124 × 56 × 33 mm and 97 × 55 × 42 mm, respectively (Figure 1a). In addition, there was splenomegaly and peritoneal effusion.

Computed tomography angiography confirmed the ultrasound findings, and other variant arteries were found (Figure 1b). Specifically, the classic celiac trunk was absent, with the left hepatic artery and left gastric artery co-trunk as the first branch originating from the anterior wall of the abdominal aorta. The common hepatic artery and splenic artery originated from the anterior wall of the aorta below the left hepatic-gastric trunk. The right hepatic artery originated from the superior mesenteric artery. There was tortuous dilatation of the esophageal and gastric fundus veins. The diagnosis was congenital hepatic vascular variations, with common hepatic artery-portal vein fistula-systemic venous shunt.

The patient was treated with fasting, hemostasis and stomach protection, blood transfusion, and portal pressure reduction with beta-blockers. After pre-operative preparation, the patient was treated with interventional embolization under digital subtraction angiography (DSA). The occluder was placed at the arterial end of the common hepatic artery-portal vein fistula (Figure 1f). The patient was discharged home 10 days after surgery, and her hemoglobin was 107 g/L. The patient was prescribed timely intake of proton pump inhibitors. She was told to rest more, avoid strenuous exercise, and go to hospital once a month for a follow-up visit. During the 3-month follow-up, the patient was in good condition. Ultrasound examination showed that the position of the occluder was normal, and the fistula gradually became smaller until it closed completely (Figure 1f).

## Discussion

Variations of the visceral vessels have important implications in cases of liver transplantation, laparoscopic surgery, abdominal radiation intervention, and abdominal trauma. In classical visceral vascular anatomy (applicable in 64–91% of the normal population), the celiac trunk originates from the anterior wall of the aorta at the level of the first lumbar vertebra, and its branches are the common hepatic artery, the left gastric artery, and the splenic artery (2, 3, 6). However, rare variation of the abdominal trunk branches occurs at rates of 8–12% (7).

In 1904, Tandler (8) provided an embryological explanation for these variations. The 4 trunks on the posterior wall of the primitive abdominal aorta in the embryonic stage are, from superior to inferior: left gastric artery, hepatic artery, splenic artery, and superior mesenteric artery. There are also longitudinal anastomotic arteries among them. During embryonic development, the 4 stems gradually rotate to the ventral side, and concomitantly, the anastomotic arteries are interrupted and separated. If the rotation is abnormally terminated or the dislocation of the anastomotic arteries is interrupted or incomplete, it will lead to variation (8).

Some scholars have classified these variations (9–11). Among them, the classifications of Michels (11) and Adachi (9) are more traditional, and serve as the benchmarks for all subsequent contributions in this field. In 1928, Adachi dissected 252 cadavers and analyzed variations of the abdominal trunk, differentiating them into six main types and 28 subtypes (9). Michels (11) classified the abdominal trunk into seven types from 200 autopsies (11). The most prevalent variations are those of the gastrosplenic trunk and hepatosplenic trunk.

The variations of the abdominal trunk branches reported in the present case were not described in either of the Adachi or Michels classifications. However, the variation found by Demirtas et al. (12) in the anatomy of a female corpse is very similar: one hepatogastric trunk originated from the front of the abdominal aorta and divides into an accessory left hepatic artery and left gastric artery. Furthermore, the other hepatosplenic trunk originated 1.5 cm below the hepatogastric trunk, and divided into the common hepatic artery and splenic artery (12).

In our case, the common hepatic artery has no branch to supply the liver, but forms an arteriovenous fistula with the portal vein, and its function is only to supply arterial blood to the fistula. The arterial supply of the liver is directly provided by the abnormal origin of the left and right hepatic arteries. The left hepatic artery and the left gastric artery originate from the anterior wall of the abdominal aorta; the right hepatic artery originates from the superior mesenteric artery.

The normal pattern of the common hepatic artery is to form the gastroduodenal and proper hepatic artery, which then divides distally into the right and left hepatic branches. The branching variations of the hepatic artery are also described in detail in the literature and anatomical monographs (13–15). Among these variations are the 10 variant subtypes of the hepatic arterial system classified by Michel (15). Variant patterns of the hepatic lobes include blood supplies from the superior mesenteric artery, left gastric artery, aorta, or other visceral branches. If these vessels are present in addition to the normal arterial supply, they are termed accessory hepatic arteries; but if they are the primary arterial supply, they are referred to as replaced hepatic arteries (13). The replaced right hepatic artery arising from the superior mesenteric artery is the best-known variation, accounting for 15% (16), while the replaced left hepatic artery originating from the left gastric artery occurs in 5.2–6.9% (16, 17). The left and right hepatic arteries directly originate from the aorta (2–4%) (18, 19).

In fact, most of the variations have little surgical significance (6). Because most arterial variations have no clinical manifestations, they are most often discovered incidentally during autopsy. However, in the current case a 12-year-old girl presented to a physician with gastrointestinal bleeding, and a congenital visceral vascular variation was found. The key disease was portal hypertension caused by congenital arteriovenous fistula, leading to the rupture and bleeding of esophageal and gastric varices. To our best knowledge, there has been no previous relevant report of a giant arteriovenous fistula formed by the common hepatic artery and portal vein. Notably, the incidence of congenital hepatic arterioportal fistula (HAPF) is <10% (20, 21).

Histologically, HAPFs can be classified as follows: type 1, small and peripheral; type 2, large and central; and type 3, intractable and congenital. Type 3 mostly occurs in the liver, and malabsorption and dysplasia are common manifestations in infancy. Older children may have severe portal hypertension, such as esophageal varices that cause upper gastrointestinal bleeding (22). The present case may be considered HAPFS type 3.

The portal vein system has large volume and small resistance, which will lead to portal hypertension if the portal vein flow or vascular resistance increases. The common hepatic artery, originated from the abdominal aorta, causes a large amount of arterial blood flow to the portal vein, and then shunts to the systemic circulation through the left branch of the portal vein. Under normal circumstances, the portal vein provides 75% of the liver input blood volume and 50% of the oxygen supply. Damage of the arterial blood supply can lead to ischemia.

In our present case, the congenital arteriovenous fistula damaged the arterial blood supply of the liver. Fortunately, the left and right liver arteries supplied the liver. At the same time, the portosystemic shunt made the patient resistant to portal hypertension before she was 12 years old, and there was no abnormal liver function and hepatic encephalopathy during this period.

Identifying a visceral vascular variation has important diagnostic and therapeutic significance, because vascular distortion directly affects the procedural strategies of abdominal surgery. This is especially relevant when variation of the hepatic artery is involved. Abnormal arterial anatomy increases the surgical complexity and potential risks of arterial supply injury, which can lead to ischemia, biliary fistula, hemorrhage, and liver abscess. Therefore, clear recognition of these arterial variations, both pre-operatively and intraoperatively, improves the probability of successful operation and limits the harmful results of complicated hepatobiliary surgery.

Because of its convenience, economy, reliability, and non-invasiveness, ultrasonography is often the first choice for imaging examination, and it provides much detailed information about vascular anatomy and hemodynamics. Computed tomography is helpful to describe shunt and identify the types of anatomical malformations. DSA can directly display the anatomical structure of blood vessels; compared with traditional open surgery, DSA can better identify multiple shunts and block the fistula at the same time. Multi-imaging examinations can provide accurate pre-operative evaluation for hepatobiliary surgery and interventional radiotherapy, help to design the best surgical plan, avoid potential catastrophic complications, and improve the success rate of donor transplantation.

## Conclusion

The present study reports an extremely rare and complicated case of portal hypertension caused by a congenital variation in the visceral vasculature. Possible anatomical variations of visceral vessels should be considered in surgical and radiological evaluations. Variations must be identified pre-operatively *via* multiple imaging examinations, to avoid intraoperative vascular injury and post-operative complications.

## Data Availability Statement

The raw data supporting the conclusions of this article will be made available by the authors, without undue reservation.

## Ethics Statement

This study was approved by the Ethics Committee of Guangdong Women and Children Hospital and written informed consent was obtained from the patient's mother. The patients/participants provided their written informed consent to participate in this study.

## Author Contributions

YW and YP designed and performed most of the investigation, data analysis, and wrote the manuscript. ZY and CY provided data curation. NS and ML contributed to interpretation of the data and analyses. All the authors read and approved the manuscript.

## Conflict of Interest

The authors declare that the research was conducted in the absence of any commercial or financial relationships that could be construed as a potential conflict of interest.

## Publisher's Note

All claims expressed in this article are solely those of the authors and do not necessarily represent those of their affiliated organizations, or those of the publisher, the editors and the reviewers. Any product that may be evaluated in this article, or claim that may be made by its manufacturer, is not guaranteed or endorsed by the publisher.

## Abbreviations

DSA: digital subtraction angiography.

## References

[1] Bergman RA Afifi AK Miyauchi R. Compendium of human anatomic variation. Urban Schwarzenberg. (1988) 24:72–5. 10.1016/0893-6080(88)90114-128368125

[2] Farghadani M Momeni M Hekmatnia A Momeni F Baradaran Mahdavi MM. Anatomical variation of celiac axis, superior mesenteric artery, and hepatic artery: evaluation with multidetector computed tomography angiography. J Res Med Sci. (2016) 21:129. 10.4103/1735-1995.196611 28331515 PMC5348823

[3] Vandamme JP Bonte J. The branches of the celiac trunk. Acta Anat (Basel). (1985) 122:110–4. 10.1159/000145991 4013640

[4] Huang Y Mu GC Qin XG Chen ZB Lin JL Zeng YJ. Study of celiac artery variations and related surgical techniques in gastric cancer. World J Gastroenterol. (2015) 21:6944–51. 10.3748/wjg.v21.i22.6944 26078572 PMC4462736

[5] Mu GC Huang Y Liu ZM Chen ZB Wu XH Qin XG. Relationship between celiac artery variation and number of lymph nodes dissection in gastric cancer surgery. World J Gastrointest Oncol. (2019) 11:499–508. 10.4251/wjgo.v11.i6.499 31236200 PMC6580319

[6] Sureka B Mittal MK Mittal A Sinha M Bhambri NK Thukral BB. Variations of celiac axis, common hepatic artery and its branches in 600 patients. Indian J Radiol Imaging. (2013) 23:223–33. 10.4103/0971-3026.120273 24347852 PMC3843330

[7] Ugurel MS Battal B Bozlar U Nural MS Tasar M Ors F. Anatomical variations of hepatic arterial system, coeliac trunk and renal arteries: an analysis with multidetector CT angiography. Br J Radiol. (2010) 83:661–7. 10.1259/bjr/21236482 20551256 PMC3473504

[8] Tandler J. Über die varietäten der arteria coeliaca und deren entwicklung. Anat Hefte. (1904) 25:472–500. 10.1007/BF02300762

[9] Adachi B. Das arteriensystem der japaner. Kaiserlich Japanischen Universität zu Kyoto Kenkysha. (1928) 2:20–71.

[10] Lipshutz B. A composite study of the coeliac axis artery. Ann Surg. (1917) 65:159–69. 10.1097/00000658-191702000-00006 17863663 PMC1426316

[11] Michels NA. Blood supply and anatomy of the upper abdominal organs with a descriptive atlas. Anat Rec. (1960) 2:153–4.

[12] Demirtas K Gulekon N Kurkcuoglu A Yildirim A Gozil R. Rare variation of the celiac trunk and related review. Saudi Med J. (2005) 26:1809–11. 16311672

[13] Hiatt JR Gabbay J Busuttil RW. Surgical anatomy of the hepatic arteries in 1000 cases. Ann Surg. (1994) 220:50–2. 10.1097/00000658-199407000-00008 8024358 PMC1234286

[14] Kamel IR Kruskal JB Pomfret EA Keogan MT Warmbrand G Raptopoulos V. Impact of multidetector CT on donor selection and surgical planning before living adult right lobe liver transplantation. AJR Am J Roentgenol. (2001) 176:193–200. 10.2214/ajr.176.1.1760193 11133565

[15] Michels NA. Newer anatomy of the liver and its variant blood supply and collateral circulation. Am J Surg. (1966) 112:337–47. 10.1016/0002-9610(66)90201-7 5917302

[16] Winston CB Lee NA Jarnagin WR Teitcher J DeMatteo RP Fong Y. CT angiography for delineation of celiac and superior mesenteric artery variants in patients undergoing hepatobiliary and pancreatic surgery. AJR Am J Roentgenol. (2007) 189:W13–19. 10.2214/AJR.04.1374 17579128

[17] De Cecco CN Ferrari R Rengo M Paolantonio P Vecchietti F Laghi A. Anatomic variations of the hepatic arteries in 250 patients studied with 64-row CT angiography. Eur Radiol. (2009) 19:2765–70. 10.1007/s00330-009-1458-7 19471940

[18] Lippert H Pabst R. Arterial Variations in Man. Munich: JF Bergman Verlag (1985). p. 30–3.

[19] Van Damme JP Bonte J. Vascular Anatomy in Abdominal Surgery. Stuttgart: Georg Thieme Verlag (1990). p. 4–42.

[20] Van Way CW III Crane JM Riddell DH Foster JH Arteriovenous fistula in the portal circulation. Surgery. (1971) 70:876–890. 5124667

[21] Zhang DY Weng SQ Dong L Shen XZ Qu XD. Portal hypertension induced by congenital hepatic arterioportal fistula: report of four clinical cases and review of the literature. World J Gastroenterol. (2015) 21:2229–35. 10.3748/wjg.v21.i7.2229 25717263 PMC4326165

[22] Guzman EA McCahill LE Rogers FB. Arterioportal fistulas: introduction of a novel classification with therapeutic implications. J Gastrointest Surg. (2006) 10:543–50. 10.1016/j.gassur.2005.06.022 16627220

